# Crystal structure of *catena*-poly[[[tetra­aqua­iron(II)]-*trans*-μ-1,2-bis­(pyridin-4-yl)ethene-κ^2^ *N*:*N*′] bis­(*p*-toluene­sulfonate) methanol disolvate]

**DOI:** 10.1107/S2056989017017054

**Published:** 2017-11-30

**Authors:** Volodymyr M. Hiiuk, Diana D. Barakhty, Sergiu Shova, Ruslan A. Polunin, Il’ya A. Gural’skiy

**Affiliations:** a Department of Chemistry, Taras Shevchenko National University of Kyiv, Volodymyrska St. 64, Kyiv 01601, Ukraine; b Faculty of Natural Sciences, National University of Kyiv-Mohyla Academy, Skovorody St. 2, Kyiv 04070, Ukraine; c UkrOrgSyntez Ltd, Schorsa St. 29, Kyiv 01133, Ukraine; d "Petru Poni" Institute Of Macromolecular Chemistry, Romanian Academy of Science, Aleea Grigore Ghica Voda 41-A, RO-700487 Iasi, Romania; e L.V. Pisarzhevskii Institute of Physical Chemistry, National Academy of Sciences of Ukraine, Prospekt Nauky 31, Kyiv 03028, Ukraine

**Keywords:** crystal structure, polymeric complex, iron(II) complex, 1,2-bis­(pyridin-4-yl)ethene, hydrogen bonding

## Abstract

In the title polymeric complex, {[Fe(C_12_H_10_N_2_)_2_(H_2_O)_4_](CH_3_C_6_H_4_SO_3_)_2_·2CH_3_OH}_*n*_, the Fe^II^ cation, located on an inversion centre, is coordinated by four water mol­ecules in the equatorial positions and two 1,2-bis­(pyridin-4-yl)ethene mol­ecules in the axial positions. This results in a distorted octa­hedral geometry for the [N_2_O_4_] coordination polyhedron. The 1,2-bis­(pyridin-4-yl)ethene mol­ecules bridge the Fe^II^ cations, forming polymeric chains running along the *a-*axis direction. Stabilization of the crystal structure is provided by O—H⋯O hydrogen bonds; these are formed by coordinated water mol­ecules as donors towards the O atoms of the methanol mol­ecules and tosyl­ate anions as acceptors of protons, leading to the formation of a three-dimensional supra­molecular network. Weak C—H⋯O hydrogen bonds are also observed in the crystal.

## Abstract 2

The 1,2-bis­(pyridin-4-yl)ethene mol­ecules bridge Fe^II^ cations to form polymeric chains running along the *a* axis.

## Chemical context   

Transition metal complexes containing pyridine or substituted pyridines as ligands are of current inter­est due to their supra­molecular arrangements and the probability of being spin-crossover compounds. Spin crossover (SCO), sometimes referred to as a spin transition or a spin equilibrium behaviour, is a phenomenon that occurs in some metal complexes wherein the spin state of a compound changes due to the influence of external stimuli such as temperature, pressure, light irradiation, magnetic field or guest effects (Gütlich & Goodwin, 2004 ▸). Bridging N-donor ligands are often used to produce Fe-based SCO complexes; for example, pyrazine is known to form inter­esting three-dimensional frameworks with remarkable transition characteristics (Muñoz & Real, 2011 ▸; Gural’skiy, Golub *et al.*, 2016 ▸; Gural’skiy, Shylin *et al.*, 2016 ▸).

A variation of the aromatic N-donor ligand can lead to possible spin-state modulation in transition metal complexes (Gütlich & Goodwin, 2004 ▸). In recent years, particular attention has been drawn to bridging ligands that are able to form analogues of Hoffman clathrates with a large pore size. These ligands include bridge-polydentate derivatives of pyridine and other azine ligands (Muñoz & Real, 2011 ▸). Importantly, Fe-based SCO in analogues of Hoffman clathrates is known in complexes with 1,2-bis­(pyridin-4-yl)ethene as a bridging N-donor ligand. Its complex with cyano­argentate as a co-ligand shows one of the largest thermal hysteresis (*ca* 95K wide) observed for spin-crossover complexes (Niel *et al.*, 2002 ▸).

Here we report on the title new polymeric compound based on 1,2-bis­(pyridin-4-yl)ethene in which Fe^II^ ions are stabilized in the high-spin state.
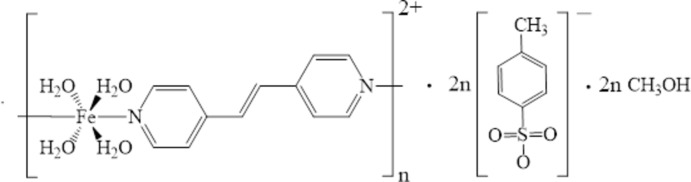


## Structural commentary   

**Figure 1:**
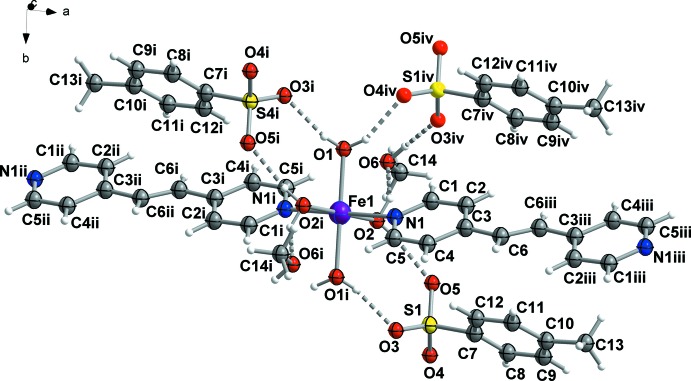
A fragment of the mol­ecular structure of the title compound showing the atom-labelling scheme. Displacement ellipsoids are drawn at the 50% probability level. [Symmetry codes: (i) −*x*, 1 − *y*, 1 − *z*; (ii) −1 + *x*, *y*, *z*; (iii) 1 − *x*, 1 − *y*, 1 − *z*; (iv) *x*, −1 + *y*, *z*.]

The Fe^II^ cation has a distorted octa­hedral coordination environment [FeN_2_O_4_], formed by two N atoms of 1,2-bis(pyridin-4-yl)ethene and by four O atoms of four water mol­ecules (Fig. 1 ▸). Two 1,2-bis­(pyridin-4-yl)ethene mol­ecules are coordinated at the axial positions [with an Fe—N distance of 2.218 (2) Å]. The equatorial positions of the Fe^II^ cation are occupied by four O-coordinated water mol­ecules with bond lengths Fe1—O1 = 2.114 (2) and Fe1—O2 = 2.077 (2) Å. The small difference in the lengths of the Fe—O bonds of 0.037 Å could be associated with a different participation of the water hydrogen atoms in hydrogen bonding. The metal-to-ligand distances clearly indicate the high-spin nature of the complex described herein.

The Fe^II^ octa­hedral distortion parameter (the sum of the moduli of the deviations from 90° for all *cis* bond angles) is Σ|90 − Θ| = 28.15 (8), where Θ is the *cis*-N—Fe—O and *cis*-O—Fe—O angles in the coordination environment of the Fe^II^ atom. This value indicates a significant polyhedral distortion, which can be explained by the Jahn–Teller effect and the presence of different types of ligands.

## Supra­molecular features   

**Figure 2:**
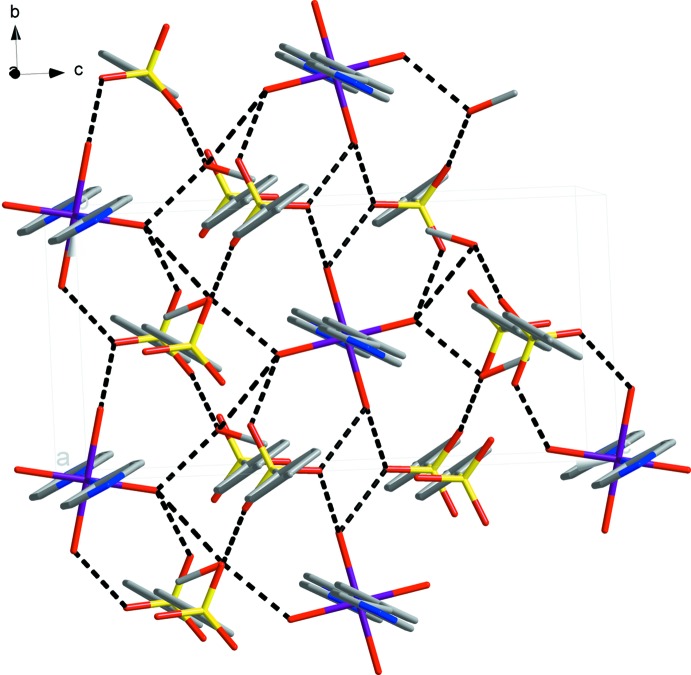
Crystal structure of the title compound, showing hydrogen bonds as dashed lines. Colour key: violet Fe, yellow S, blue N, grey C and red O.

**Figure 3:**
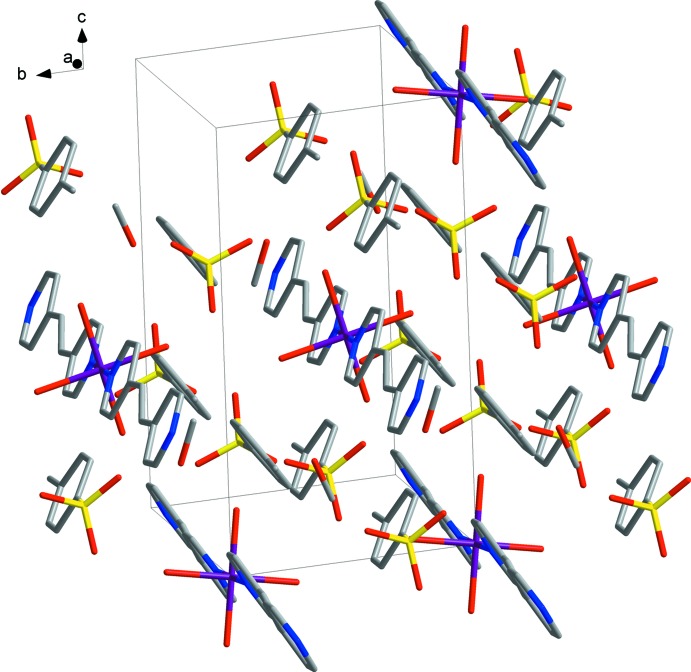
The crystal packing. Colour key: violet Fe, yellow S, blue N, grey C and red O.

The coordination structure is formed by binding 1,2-bis(pyridin-4-yl)ethene fragments with Fe^II^ cations into polymer chains that propagate along the *a*-axis direction. Stabilization in the crystal structure is ensured by O—H⋯O hydrogen bonds (Fig. 2 ▸, Table 1 ▸): (i) H atoms of water mol­ecules and the oxygen atoms of tosyl­ate anions; (ii) H atoms of water mol­ecules and methanol mol­ecules; (iii) H atoms of the hydroxyl group of methanol with the tosyl­ate anion. The compound contains two solvate mol­ecules of methanol per Fe^II^ cation. In the crystal lattice, each tosyl­ate anion is connected with three water mol­ecules of the complex cation, leading to the formation of a three-dimensional supra­molecular network (Fig. 2 ▸). In addition, weak C—H⋯O hydrogen bonds are also observed in the crystal. A view of the packing is shown in Fig. 3 ▸.

## Database survey   

A survey of the Cambridge Structural Database confirmed that the structure of the title complex has not been reported previously. 41 structures are known with an Fe cation coord­inated by four water O atoms and two N atoms from the pyridine fragment. The survey yielded the structure of one related compound, in which the Fe^II^ cation has a distorted octa­hedral coordination environment [FeN_2_O_4_], formed by two N atoms of 1,2-bis­(pyridin-4-yl)ethene and by four O atoms of four water mol­ecules; however, it contains 2,6-dioxo-1,2,3,6-tetra­hydro­pyrimidin-4-olate as the anion and crystallizes in the ortho­rhom­bic *Pbcn* space group. In this analogue, Fe1—N1 = 2.2304 (2), Fe1—O2 = 2.1030 (2) and Fe1—O4 = 2.0908 (2) Å (Garcia *et al.*, 2011 ▸), contrary to what is observed in the title compound.

## Synthesis and crystallization   

Crystals of the title compound were prepared by the slow diffusion method between three layers in a 10 ml tube. The first layer was a solution of [Fe(OTs)_2_]·6H_2_O (OTs = *p*-toluene­sulfonate) (0.1012 g, 0.02 mmol) in water (2.5 ml), the second was a mixture of water/methanol (1:1, 5 ml) and the third layer was a solution of 1,2-bis­(pyridin-4-yl)ethene (0.0352 g, 0.02 mmol) in methanol (2.5 ml). After two weeks, red crystals grew in the second layer; these were collected and maintained under the mother solution until measured.

## Refinement   

Crystal data, data collection and structure refinement details are summarized in Table 2 ▸. All aromatic hydrogens and hydrogen atoms of the CH groups were placed in their expected calculated positions (C—H = 0.95 Å) and refined as riding with *U*_iso_(H) = 1.2*U*_iso_(C). Methyl H atoms were placed in their expected calculated positions (C—H = 0.98 Å) and refined as rotating groups with *U*_iso_(H) = 1.5*U*_eq_(C). Hydrogen atoms of the water mol­ecules were assigned based on the difference-Fourier map, and the O—H distances and the H—O—H angles were constrained using DFIX (O—H = 0.84 Å) and DANG (H—H = 1.34 Å) instructions. The hydrogen H atom of the solvent methanol mol­ecule was assigned based on the difference-Fourier map, and the O—H distance was constrained using a DFIX (O—H = 0.96 Å) instruction.

## Supplementary Material

Crystal structure: contains datablock(s) global, I. DOI: 10.1107/S2056989017017054/xu5910sup1.cif

Structure factors: contains datablock(s) I. DOI: 10.1107/S2056989017017054/xu5910Isup2.hkl

CCDC reference: 1587771

Additional supporting information:  crystallographic information; 3D view; checkCIF report

## Figures and Tables

**Table 1:** Hydrogen-bond geometry (Å, °)

*D*—H⋯*A*	*D*—H	H⋯*A*	*D*⋯*A*	*D*—H⋯*A*
O1—H1*A*⋯O3^i^	0.83 (2)	1.93 (2)	2.753 (3)	172 (3)
O1—H1*B*⋯O3^ii^	0.84 (2)	1.90 (2)	2.726 (2)	168 (3)
O2—H2*A*⋯O6	0.84 (2)	1.82 (2)	2.654 (3)	172 (3)
O2—H2*B*⋯O5	0.84 (2)	1.92 (2)	2.752 (3)	170 (3)
O6—H6*A*⋯O4^ii^	0.91 (2)	1.95 (2)	2.823 (3)	161 (3)
C4—H4⋯O5^iii^	0.95	2.51	3.406 (3)	157
C13—H13*B*⋯O4^iv^	0.98	2.59	3.562 (4)	172
C13—H13*C*⋯O5^v^	0.98	2.51	3.465 (4)	165

Symmetry codes: (i) 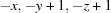; (ii) 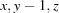; (iii) 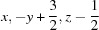; (iv) 
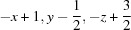
; (v) 
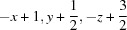
.

**Table 2:** Experimental details

Crystal data	
Chemical formula	[Fe(C_12_H_10_N_2_)(H_2_O)_4_](C_7_H_7_O_3_S)_2_·2CH_4_O
*M*_r_	716.59
Crystal system, space group	Monoclinic, *P*2_1_/*c*
Temperature (K)	200
*a*, *b*, *c* (Å)	13.8416 (9), 7.7686 (4), 16.4076 (13)
β (°)	111.845 (9)
*V* (Å^3^)	1637.6 (2)
*Z*	2
Radiation type	Mo *K*α
μ (mm^−1^)	0.65
Crystal size (mm)	0.35 × 0.2 × 0.15

Data collection	
Diffractometer	Rigaku OD Xcalibur, Eos
Absorption correction	Multi-scan (*CrysAlis PRO*; Rigaku OD, 2015 ▸)
*T*_min_, *T*_max_	0.920, 1.000
No. of measured, independent and observed [*I* > 2σ(*I*)] reflections	6379, 2857, 2363
*R*_int_	0.030
(sin θ/λ)_max_ (Å^−1^)	0.595

Refinement	
*R*[*F* ^2^ > 2σ(*F* ^2^)], *wR*(*F* ^2^), *S*	0.041, 0.097, 1.06
No. of reflections	2857
No. of parameters	227
No. of restraints	7
H-atom treatment	H atoms treated by a mixture of independent and constrained refinement
Δρ_max_, Δρ_min_ (e Å^−3^)	0.24, −0.33

Computer programs: *CrysAlis PRO* (Rigaku OD, 2015 ▸), *SHELXS97* (Sheldrick, 2008 ▸), *SHELXL2017* (Sheldrick, 2015 ▸) and *OLEX2* (Dolomanov *et al.*, 2009 ▸).

## supplementary crystallographic information

### Crystal data

**Table:**

[Fe(C_12_H_10_N_2_)(H_2_O)_4_](C_7_H_7_O_3_S)_2_·2CH_4_O	*F*(000) = 752
*M**_r_* = 716.59	*D*_x_ = 1.453 Mg m^−^^3^
Monoclinic, *P*2_1_/*c*	Mo *K*α radiation, λ = 0.71073 Å
*a* = 13.8416 (9) Å	Cell parameters from 2979 reflections
*b* = 7.7686 (4) Å	θ = 2.6–31.1°
*c* = 16.4076 (13) Å	µ = 0.65 mm^−^^1^
β = 111.845 (9)°	*T* = 200 K
*V* = 1637.6 (2) Å^3^	Prism, clear intense red
*Z* = 2	0.35 × 0.2 × 0.15 mm

### Data collection

**Table:**

Rigaku OD Xcalibur, Eos diffractometer	2363 reflections with *I* > 2σ(*I*)
Detector resolution: 8.0797 pixels mm^-1^	*R*_int_ = 0.030
ω scans	θ_max_ = 25.0°, θ_min_ = 2.6°
Absorption correction: multi-scan (CrysAlis PRO; Rigaku OD, 2015)	*h* = −16→16
*T*_min_ = 0.920, *T*_max_ = 1.000	*k* = −8→9
6379 measured reflections	*l* = −19→11
2857 independent reflections	

### Refinement

**Table:**

Refinement on *F*^2^	Primary atom site location: structure-invariant direct methods
Least-squares matrix: full	Hydrogen site location: mixed
*R*[*F*^2^ > 2σ(*F*^2^)] = 0.041	H atoms treated by a mixture of independent and constrained refinement
*wR*(*F*^2^) = 0.097	*w* = 1/[σ^2^(*F*_o_^2^) + (0.0396*P*)^2^ + 0.6283*P*] where *P* = (*F*_o_^2^ + 2*F*_c_^2^)/3
*S* = 1.06	(Δ/σ)_max_ < 0.001
2857 reflections	Δρ_max_ = 0.24 e Å^−^^3^
227 parameters	Δρ_min_ = −0.32 e Å^−^^3^
7 restraints	

### Special details

**Table:**

Geometry. All esds (except the esd in the dihedral angle between two l.s. planes) are estimated using the full covariance matrix. The cell esds are taken into account individually in the estimation of esds in distances, angles and torsion angles; correlations between esds in cell parameters are only used when they are defined by crystal symmetry. An approximate (isotropic) treatment of cell esds is used for estimating esds involving l.s. planes.

### Fractional atomic coordinates and isotropic or equivalent isotropic displacement parameters (Å^2^)

**Table:**

	*x*	*y*	*z*	*U*_iso_*/*U*_eq_	
Fe1	0.000000	0.500000	0.500000	0.01832 (16)	
O2	0.07337 (15)	0.5517 (3)	0.63317 (12)	0.0295 (5)	
O1	−0.00268 (15)	0.2362 (2)	0.53062 (14)	0.0279 (4)	
O6	0.06975 (17)	0.3317 (3)	0.75578 (14)	0.0403 (5)	
N1	0.15498 (16)	0.4896 (2)	0.48932 (14)	0.0218 (5)	
C1	0.23952 (19)	0.4220 (3)	0.55153 (18)	0.0243 (6)	
H1	0.231285	0.368884	0.600701	0.029*	
C3	0.3528 (2)	0.5005 (3)	0.47719 (17)	0.0235 (6)	
C2	0.3375 (2)	0.4249 (3)	0.54841 (18)	0.0269 (6)	
H2	0.394535	0.375488	0.594749	0.032*	
C4	0.2643 (2)	0.5672 (3)	0.41120 (17)	0.0256 (6)	
H4	0.269716	0.617840	0.360394	0.031*	
C6	0.4539 (2)	0.5179 (3)	0.46934 (18)	0.0251 (6)	
H6	0.453831	0.558952	0.414775	0.030*	
C5	0.1695 (2)	0.5594 (3)	0.41982 (17)	0.0246 (6)	
H5	0.110758	0.606167	0.374029	0.029*	
C14	0.0849 (3)	0.3932 (4)	0.8412 (2)	0.0470 (8)	
H14A	0.159070	0.415178	0.873617	0.071*	
H14B	0.060297	0.306700	0.872498	0.071*	
H14C	0.045634	0.500216	0.836491	0.071*	
S1	0.22227 (5)	0.97890 (8)	0.66335 (4)	0.02490 (18)	
O3	0.14978 (14)	0.9925 (2)	0.57179 (12)	0.0310 (5)	
O5	0.21658 (15)	0.8109 (2)	0.70032 (13)	0.0336 (5)	
O4	0.21348 (15)	1.1211 (2)	0.71723 (13)	0.0362 (5)	
C7	0.3476 (2)	0.9925 (3)	0.65937 (17)	0.0225 (6)	
C8	0.4272 (2)	1.0780 (3)	0.72377 (17)	0.0283 (6)	
H8	0.413993	1.136967	0.769296	0.034*	
C11	0.4661 (2)	0.9110 (3)	0.59080 (19)	0.0312 (7)	
H11	0.478876	0.854123	0.544533	0.037*	
C9	0.5267 (2)	1.0775 (3)	0.72171 (18)	0.0309 (7)	
H9	0.581277	1.136606	0.766118	0.037*	
C12	0.3668 (2)	0.9108 (3)	0.59167 (18)	0.0298 (6)	
H12	0.311753	0.855117	0.546108	0.036*	
C10	0.5476 (2)	0.9924 (3)	0.65615 (19)	0.0292 (6)	
C13	0.6567 (2)	0.9835 (4)	0.6563 (2)	0.0413 (8)	
H13A	0.653625	0.983917	0.595625	0.062*	
H13B	0.690467	0.877556	0.685580	0.062*	
H13C	0.696653	1.083302	0.687642	0.062*	
H2A	0.078 (3)	0.485 (3)	0.6748 (17)	0.061 (12)*	
H2B	0.113 (2)	0.633 (3)	0.6576 (18)	0.042 (9)*	
H1A	−0.0508 (17)	0.176 (3)	0.4972 (17)	0.043 (10)*	
H1B	0.0490 (17)	0.171 (3)	0.5494 (19)	0.043 (9)*	
H6A	0.120 (2)	0.256 (4)	0.756 (2)	0.061 (11)*	

### Atomic displacement parameters (Å^2^)

**Table:**

	*U*^11^	*U*^22^	*U*^33^	*U*^12^	*U*^13^	*U*^23^
Fe1	0.0129 (3)	0.0212 (3)	0.0211 (3)	−0.0002 (2)	0.0065 (2)	0.0006 (2)
O2	0.0277 (11)	0.0355 (11)	0.0217 (11)	−0.0106 (9)	0.0052 (9)	−0.0011 (9)
O1	0.0181 (10)	0.0212 (10)	0.0383 (12)	0.0007 (8)	0.0035 (9)	0.0010 (9)
O6	0.0434 (13)	0.0432 (12)	0.0396 (13)	0.0106 (10)	0.0215 (11)	0.0085 (10)
N1	0.0183 (11)	0.0229 (11)	0.0252 (12)	−0.0008 (9)	0.0092 (10)	0.0004 (9)
C1	0.0196 (14)	0.0281 (14)	0.0271 (14)	0.0022 (11)	0.0108 (12)	0.0068 (11)
C3	0.0218 (14)	0.0230 (13)	0.0281 (15)	−0.0009 (11)	0.0119 (12)	−0.0046 (11)
C2	0.0170 (13)	0.0324 (14)	0.0296 (16)	0.0036 (11)	0.0068 (12)	0.0071 (12)
C4	0.0247 (14)	0.0309 (14)	0.0246 (14)	−0.0020 (11)	0.0130 (12)	−0.0002 (11)
C6	0.0231 (13)	0.0281 (14)	0.0296 (15)	−0.0007 (11)	0.0161 (12)	−0.0003 (12)
C5	0.0176 (13)	0.0307 (14)	0.0248 (15)	0.0017 (11)	0.0071 (12)	0.0025 (12)
C14	0.051 (2)	0.052 (2)	0.047 (2)	−0.0027 (17)	0.0281 (18)	0.0034 (16)
S1	0.0210 (4)	0.0225 (3)	0.0289 (4)	0.0001 (3)	0.0066 (3)	−0.0020 (3)
O3	0.0209 (10)	0.0340 (10)	0.0309 (11)	0.0040 (8)	0.0014 (9)	0.0018 (9)
O5	0.0293 (11)	0.0295 (10)	0.0376 (12)	−0.0057 (9)	0.0074 (9)	0.0040 (9)
O4	0.0340 (11)	0.0359 (11)	0.0415 (12)	0.0006 (9)	0.0173 (10)	−0.0112 (9)
C7	0.0233 (14)	0.0162 (12)	0.0241 (14)	0.0016 (10)	0.0043 (11)	0.0009 (10)
C8	0.0286 (15)	0.0254 (14)	0.0272 (15)	−0.0014 (11)	0.0062 (13)	−0.0059 (12)
C11	0.0314 (16)	0.0286 (14)	0.0364 (17)	0.0003 (12)	0.0158 (14)	−0.0047 (12)
C9	0.0227 (15)	0.0274 (14)	0.0328 (16)	−0.0051 (11)	−0.0011 (13)	−0.0023 (12)
C12	0.0238 (15)	0.0287 (14)	0.0320 (16)	−0.0025 (12)	0.0048 (13)	−0.0089 (12)
C10	0.0263 (15)	0.0227 (13)	0.0351 (16)	0.0013 (11)	0.0073 (13)	0.0046 (12)
C13	0.0247 (16)	0.0435 (18)	0.053 (2)	−0.0018 (13)	0.0116 (15)	0.0061 (15)

### Geometric parameters (Å, º)

**Table:**

Fe1—O1	2.1135 (18)	C6—H6	0.9500
Fe1—O1^i^	2.1135 (18)	C5—H5	0.9500
Fe1—O2	2.0773 (19)	C14—H14A	0.9800
Fe1—O2^i^	2.0773 (19)	C14—H14B	0.9800
Fe1—N1^i^	2.218 (2)	C14—H14C	0.9800
Fe1—N1	2.218 (2)	S1—O3	1.4673 (19)
O2—H2A	0.839 (17)	S1—O5	1.4535 (19)
O2—H2B	0.839 (17)	S1—O4	1.4482 (19)
O1—H1A	0.832 (17)	S1—C7	1.764 (3)
O1—H1B	0.837 (17)	C7—C8	1.380 (4)
O6—C14	1.421 (4)	C7—C12	1.387 (4)
O6—H6A	0.910 (18)	C8—H8	0.9500
N1—C1	1.341 (3)	C8—C9	1.391 (4)
N1—C5	1.344 (3)	C11—H11	0.9500
C1—H1	0.9500	C11—C12	1.380 (4)
C1—C2	1.376 (3)	C11—C10	1.386 (4)
C3—C2	1.393 (4)	C9—H9	0.9500
C3—C4	1.398 (4)	C9—C10	1.382 (4)
C3—C6	1.459 (4)	C12—H12	0.9500
C2—H2	0.9500	C10—C13	1.511 (4)
C4—H4	0.9500	C13—H13A	0.9800
C4—C5	1.372 (3)	C13—H13B	0.9800
C6—C6^ii^	1.327 (5)	C13—H13C	0.9800
			
O2—Fe1—O2^i^	180.00 (11)	N1—C5—C4	123.8 (2)
O2^i^—Fe1—O1^i^	88.98 (8)	N1—C5—H5	118.1
O2^i^—Fe1—O1	91.02 (8)	C4—C5—H5	118.1
O2—Fe1—O1^i^	91.01 (8)	O6—C14—H14A	109.5
O2—Fe1—O1	88.98 (8)	O6—C14—H14B	109.5
O2^i^—Fe1—N1	91.06 (8)	O6—C14—H14C	109.5
O2^i^—Fe1—N1^i^	88.94 (8)	H14A—C14—H14B	109.5
O2—Fe1—N1^i^	91.06 (8)	H14A—C14—H14C	109.5
O2—Fe1—N1	88.94 (8)	H14B—C14—H14C	109.5
O1^i^—Fe1—O1	180.0	O3—S1—C7	105.38 (12)
O1—Fe1—N1	94.96 (7)	O5—S1—O3	111.68 (11)
O1—Fe1—N1^i^	85.04 (7)	O5—S1—C7	106.02 (11)
O1^i^—Fe1—N1^i^	94.96 (7)	O4—S1—O3	112.92 (11)
O1^i^—Fe1—N1	85.04 (7)	O4—S1—O5	113.58 (12)
N1—Fe1—N1^i^	180.0	O4—S1—C7	106.52 (12)
Fe1—O2—H2A	127 (2)	C8—C7—S1	121.1 (2)
Fe1—O2—H2B	129 (2)	C8—C7—C12	119.8 (3)
H2A—O2—H2B	104 (3)	C12—C7—S1	119.1 (2)
Fe1—O1—H1A	118 (2)	C7—C8—H8	120.2
Fe1—O1—H1B	126 (2)	C7—C8—C9	119.7 (3)
H1A—O1—H1B	105 (3)	C9—C8—H8	120.2
C14—O6—H6A	113 (2)	C12—C11—H11	119.4
C1—N1—Fe1	123.36 (17)	C12—C11—C10	121.3 (3)
C1—N1—C5	116.1 (2)	C10—C11—H11	119.4
C5—N1—Fe1	120.44 (17)	C8—C9—H9	119.5
N1—C1—H1	118.0	C10—C9—C8	121.1 (3)
N1—C1—C2	123.9 (2)	C10—C9—H9	119.5
C2—C1—H1	118.0	C7—C12—H12	120.1
C2—C3—C4	116.3 (2)	C11—C12—C7	119.7 (3)
C2—C3—C6	124.3 (2)	C11—C12—H12	120.1
C4—C3—C6	119.4 (2)	C11—C10—C13	120.3 (3)
C1—C2—C3	119.9 (2)	C9—C10—C11	118.3 (3)
C1—C2—H2	120.1	C9—C10—C13	121.3 (3)
C3—C2—H2	120.1	C10—C13—H13A	109.5
C3—C4—H4	120.0	C10—C13—H13B	109.5
C5—C4—C3	120.0 (2)	C10—C13—H13C	109.5
C5—C4—H4	120.0	H13A—C13—H13B	109.5
C3—C6—H6	116.7	H13A—C13—H13C	109.5
C6^ii^—C6—C3	126.6 (3)	H13B—C13—H13C	109.5
C6^ii^—C6—H6	116.7		

Symmetry codes: (i) −*x*, −*y*+1, −*z*+1; (ii) −*x*+1, −*y*+1, −*z*+1.

### Hydrogen-bond geometry (Å, º)

**Table:**

*D*—H···*A*	*D*—H	H···*A*	*D*···*A*	*D*—H···*A*
O1—H1*A*···O3^i^	0.83 (2)	1.93 (2)	2.753 (3)	172 (3)
O1—H1*B*···O3^iii^	0.84 (2)	1.90 (2)	2.726 (2)	168 (3)
O2—H2*A*···O6	0.84 (2)	1.82 (2)	2.654 (3)	172 (3)
O2—H2*B*···O5	0.84 (2)	1.92 (2)	2.752 (3)	170 (3)
O6—H6*A*···O4^iii^	0.91 (2)	1.95 (2)	2.823 (3)	161 (3)
C4—H4···O5^iv^	0.95	2.51	3.406 (3)	157
C13—H13*B*···O4^v^	0.98	2.59	3.562 (4)	172
C13—H13*C*···O5^vi^	0.98	2.51	3.465 (4)	165

Symmetry codes: (i) −*x*, −*y*+1, −*z*+1; (iii) *x*, *y*−1, *z*; (iv) *x*, −*y*+3/2, *z*−1/2; (v) −*x*+1, *y*−1/2, −*z*+3/2; (vi) −*x*+1, *y*+1/2, −*z*+3/2.
